# Numerical study on the influence of flexible net permeability on debris flow impact loads

**DOI:** 10.1371/journal.pone.0325364

**Published:** 2025-07-11

**Authors:** Yanfen Wang, Siyu Xiao, Linbo Qiao

**Affiliations:** 1 School of Civil Engineering and Urban Planing, Liupanshui Normal University, Liupanshui, China; 2 Guizhou Provincial Mountainous Expressway Intelligent Operation and Maintenance Engineering Research Center, Guizhou Expressway Group, Guiyang, China; Albany Museum, SOUTH AFRICA

## Abstract

Debris flows in mountainous regions pose a significant threat to human lives and property. Flexible protection nets are widely used in debris flow prevention projects; however, their effectiveness requires improvement. This study integrates field investigations, DEM simulations, and numerical analyses to comprehensively explore the impact of flexible net permeability on debris flow dynamics. The results indicate that debris flow particle size significantly affects impact velocity, with larger particles exhibiting higher velocities. Different accumulation patterns also lead to distinct velocity distributions. Regarding the tensile forces in the flexible net’s support ropes, the lower ropes bear greater loads, and these forces increase as debris flow particle size grows. Debris flow accumulation height is influenced by particle size and deposition patterns; excessive heights can damage the nets. Small debris flows cause minimal net deformation, while large debris flows result in more substantial deformation due to full interception. These findings provide critical insights for optimizing flexible net design in debris flow protection, guiding parameter selection and structural enhancements. Additionally, they inform the development of more effective prevention strategies, ultimately reducing disaster-related losses.

## 1. Introduction

The unique geological conditions and complex and changeable natural environments in mountainous areas interact with each other, posing many challenges to the stability of slopes and mountains, making them highly susceptible to collapses and landslides [1,2]. Once the conditions are right (such as heavy rain, engineering disturbances, etc.), they may suddenly become unstable, resulting in debris flows, which pose a significant threat to the lives and property of people in mountainous areas [3]. With the development of the social economy and the expansion of human activities, research on and prevention of debris flows have become increasingly urgent [4]. Flexible nets are flexible structures with strong deformation capabilities and diverse structural forms. They are convenient to maintain and install and play a crucial role in debris flow prevention. They can protect people’s safety, buildings, infrastructure, and ecological environment; reduce secondary disasters; and improve disaster prevention and mitigation capabilities, which is highly important for reducing the losses and impacts caused by debris flow disasters [5]. Therefore, deeply exploring the mechanical mechanisms and protection effectiveness of flexible nets during the process of withstanding debris flow impacts is crucial. In particular, studying the important parameter of flexible net permeability is highly important for understanding how it affects debris flow impact loads. Through accurate numerical simulations and analyses, clarifying the mechanical response laws of flexible nets with different permeabilities when facing various debris flow impacts can provide a solid theoretical basis for the optimized design of flexible nets, thereby promoting the technological progress of debris flow prevention and control projects and minimizing the losses caused by debris flow disasters.

In basic research on landslides and debris flows, Hungr et al. (2014) updated the classification of landslides, providing a standard framework for accurately identifying and studying different types of landslides and potential debris flows [6]. Crosta et al. (2016) comprehensively reviewed the susceptibility, hazard, and risk assessment of landslides, which is helpful for obtaining a deeper understanding of the occurrence probability and potential impact range of landslides and debris flow disasters and provides macrolevel guidance for formulating prevention and control strategies [2]. Griffiths et al. (2017) used limit equilibrium and finite element analysis methods to study slope stability, providing theoretical and calculation methods for analysing the conditions for slope instability and debris flow generation [7]. Potyondy et al. (2004) established a bonded-particle model for rock, providing a basis for simulating the mechanical behavior of rock particles in debris flows and helping to understand the formation and movement mechanisms of debris flows from a microscopic perspective [8].

In terms of research methods, the discrete element method (DEM) and the finite element method (FEM) are widely used in the study of the interaction between debris flows and protection structures. Albaba et al. (2017) used the discrete element method to analyse the energy dissipation mechanism of debris flow impacts in detail and reported that the collision and friction between particles are among the main reasons for the energy dissipation of debris flow impacts, providing theoretical support for understanding the physical nature of the debris flow impact process [9]. Song et al. (2018) conducted experimental research on the energy dissipation of debris flow impacts through geotechnical centrifuge experiments combined with the discrete element method, verifying the reliability of discrete element simulations [10]. Liu et al. (2019) studied debris flow impacts via a coupled discrete element and finite element method, combining the advantages of these two methods. This approach can not only consider the interactions between particles but also simulate the overall mechanical response of the structure, more comprehensively revealing the complex process of the interaction between debris flows and protection structures [11]. Kwan et al. (2019) studied the impact of debris flows on flexible barriers via the finite element method, analysed the influence of different material and structural parameters on the protection effect and provided a theoretical basis for the design of flexible protection structures [12]. Research on the impact force of debris flows via the discrete element method provides important references for subsequent analyses of the impact loads on flexible nets [3,13]. In the research of flexible net protection mechanisms, significant progress has also been made. Xiao et al. (2019) studied the process of debris flow impacts on flexible nets through discrete element simulations and analysed the mechanical responses of flexible nets under different working conditions [14]. Xiao et al. (2022) further explored the influence of flexible net structures on the impact mechanical characteristics of debris flows and reported that a reasonable net structure design can effectively improve the protection performance of flexible nets [15]. Wu et al. (2022) established a simplified mechanical model of flexible protection nets under debris flow impacts, providing a convenient method for quickly evaluating the protection effect of flexible nets in practical engineering [16]. Wang et al. (2016) analysed the influencing factors of debris flow impact pressure on flexible barriers, providing ideas for understanding the influencing factors of impact loads on flexible nets [17]. Li et al. (2017) experimentally studied the energy dissipation of flexible barriers under debris flow impacts, providing experimental evidence for optimizing the energy dissipation capacity of flexible nets [18]. Zhao et al revealed the adverse effects of rock mechanics deterioration and crack propagation on slopes [19–22]. Numerical simulations of the interaction between debris flows and flexible protection structures have further enriched the understanding of the interaction process between the two [23].

In research related to practical engineering applications, the numerical analysis of the performance of flexible shed–tunnels under rock–fall impacts, although the research object is different from debris flow impacts on flexible nets, the analysis methods of the mechanical responses of structures under impact loads have certain reference significance [24,25]. Zhan et al. (2017) established an empirical prediction model for the motion distance of gully type landslides and debris flows and analysed the mechanical response mechanism of flexible nets, providing a practical method for predicting the impact range of debris flows and evaluating the protection effect of flexible nets [26]. Qi et al. (2018) experimentally studied the mechanical response process of passive flexible nets under rock–fall impacts, clarifying the key factors leading to the failure of the protection net system, which has important implications for improving the reliability of flexible nets under debris flow impacts [27]. Liu et al. (2016) studied the stress conditions of passive flexible protection nets under rock-fall impacts through experiments and numerical analyses, providing practical mechanical property data for the design of flexible nets [28]. The permeability of flexible nets affects the impact load of debris flow through the variation of rock permeability and fracture behavior [29–32]. Liu et al. (2017) proposed design countermeasures for debris–flow flexible protection systems from the perspectives of experiments and numerical analyses, providing specific guidance for the design of flexible nets in practical engineering [33]. Zheng et al. (2018) systematically studied the long-distance movement distance of landslides and debris flows, which helps to assess the hazard range of debris flows more accurately and provides a basis for the rational layout of flexible net protection structures [34].

In the research of particle motion and related measurement technologies, Yang et al. (2018) introduced in detail the particle motion characteristics and measurement methods in dense granular systems, providing technical support for studying the motion of particles in debris flows and helping to more accurately simulate and analyse the process of debris flow impacts on flexible nets [35]. In the research of flexible net material properties, Zhang et al. (2022) explored the damage mechanical behavior of flexible ring-shaped mesh sheets under repeated impact loads through experiments and finite element analyses, which is highly important for evaluating the durability of flexible nets under long-term debris flow impacts [36].

In summary, although certain research progress has been made in the field of debris flow protection, in the face of complex problems in practical engineering and increasing protection requirements, a large amount of research is still needed. Therefore, this paper uses DEM technology to study the accumulation patterns of debris flows with different flow states when they impact flexible nets, the mechanism of how the permeability of flexible nets affects the debris flow accumulation process, and the spatiotemporal variation laws of debris flow impact loads during different accumulation processes. This research integrates DEM numerical simulations with multi-scale field investigation data to systematically elucidate the dynamic regulatory mechanisms governing permeability characteristics of flexible protective nets under debris flow impact loading.

## 2. Research methods

### 2.1. Discrete element method

The discrete element method (DEM) is a numerical calculation method used to simulate and analyse the behavior of discrete particle systems. In the DEM, the particles in a continuous medium are regarded as discrete units, and each particle has its own properties, such as mass, shape, size, elastic modulus, and friction coefficient. Particles can come into contact and interact with each other, and these interactions include elastic collisions, friction, and adhesion. The DEM simulates the dynamic behavior of particles by calculating these contact forces. The motion of particles is predicted by solving Newton’s equations of motion.

Some scholars’ research is based on the Hertz–Mindlin bonding model to simulate flexible nets and the Hertz–Mindlin (no-slip) model to simulate debris flows in a discrete-element model of debris flows impacting flexible nets [18]. Through a simulation of debris flows impacting flexible nets, the influence mechanism of flexible net permeability on impact loads under debris flow impacts is studied.

### 2.2. Model establishment

#### 2.2.1. Parameter selection.

**Fig 1:**
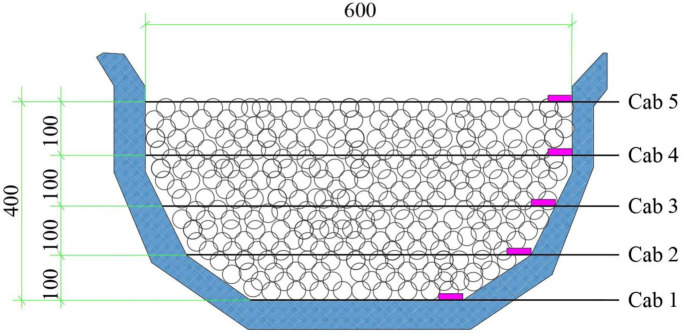
Flexible net protection structure (dimension unit: mm).

In this work, a flexible net structure with transverse support ropes is selected. The transverse support ropes are numbered from cab1 to cab5, with an interval of 0.1 m. The height of the protective net is 0.4 m, and the maximum protective width is 0.6 m. The protective structure is shown in Fig 1. The diameter of the support ropes is 5 mm, and the diameter of the mesh holes is 31.6 mm.

The elastic modulus of the support ropes is 1550 MPa. Xiao Siyou (2022) calibrated the discrete element parameters of debris flows and flexible nets on the basis of the Hertz–Mindlin bonding model and the no-slip Hertz–Mindlin model for the simulation and analysis of debris flows impacting flexible net structures [15]. The calibration of DEM simulation parameters is a critical step to ensure the accuracy of numerical results. This process begins by defining target mechanical behaviors based on laboratory data (e.g., elastic modulus, friction angle). Establish theoretical linkages between micro-scale parameters and macro-scale material properties. Sensitivity analysis is employed to evaluate parameter impacts on simulation outcomes, followed by iterative optimization using algorithms (e.g., genetic algorithms) to align simulated stress-strain responses or deformation patterns with experimental data. The accuracy of the parameter settings is a prerequisite for ensuring that the conclusions drawn from the simulation results are consistent with the actual situation. To ensure the accuracy of the simulation results, the parameters are directly quoted here without further research on parameter calibration. The simulation material parameters are shown in Table 1, the discrete element contact parameters of the debris flows and flexible nets are shown in Table 2, and the bonding parameters of the flexible net model are shown in Table 3.

**Table 1:** Simulation material parameters.

Material parameters	Poisson’s ratio	Density (kg/m^3^)	Shear modulus	Radius (mm)
Debris particles	0.2	2500	1e10	26/13/6.5
Support rope particles	0.15	7900	1e9	2.5
Mesh sheet particles	0.15	7900	1e9	1.5
Fixed particles	0.15	1e15	1e10	2.5
Chute	0.2	2500	1e8	/

**Table 2:** Model contact parameters.

Contact particles	Static friction coefficient	Dynamic friction coefficient	Recovery coefficient	Contact radius/mm
Debris – debris	0.6	0.2	0.15	0.15
Support rope – support rope	0.5	0.1	0.1	0.029
Support rope – mesh sheet	0.5	0.1	0.1	0.029
Mesh sheet – mesh sheet	0.5	0.1	0.1	0.014
Debris – chute	0.6	0.2	0.2	/
Fixed end – fixed end	0.5	0.01	0.15	/

**Table 3:** Bonding parameters.

Contact particles	Normal stiffness per square meter (N/m^3^)	Tangential stiffness per square meter (N/m^3^)	Critical tangential stress (Pa)
Support rope – support rope	5e10	4e10	3e12
Support rope – mesh sheet	5e10	4e10	3e12
Support rope – fixed particles	5e10	4e10	3e12
Mesh sheet – mesh sheet	5e11	4e11	3e12

#### 2.2.2. Working condition setting.

The flexible net is arranged at the gully entrance and fixed on both sides of the gully through fixed particles. To reduce the impact of deformation at both ends on the experiment, the mass and bonding stiffness of the fixed particles are set to be relatively large. A fixed mass can ensure this. The transverse support ropes cab1 - cab5 are set as the main load-bearing structures. The Cab1 support rope is fixed on the ground, and the energy absorber is simplified by setting fixed particles with different bonding mechanical parameters. The slope angles are set to 40°, 45°, and 50°. The purpose of setting different slope angles is to conduct comparative experiments. The three particle radii studied in this paper are 6.5 mm, 13 mm, and 26 mm. The mesh diameters are 2.4, 1.2, and 0.6 times the particle radii to change the permeability of the net and the fluidity of the debris flow.

**Fig 2:**
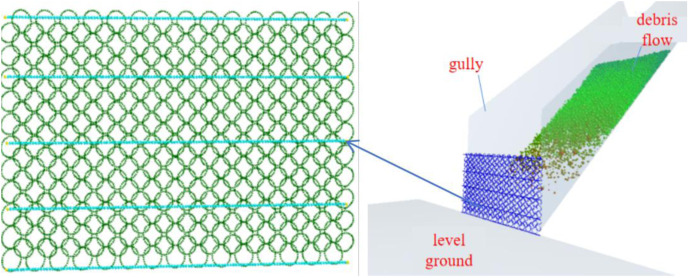
DEM model diagram of debris flows impacting flexible nets.

A debris flow with a mass of 72 kg impacts the passive flexible net. The number of support rope particles is 605, the number of fixed particles is 10, the number of mesh sheet particles is 9900, and the radius of the net is 15.8 mm. For different slope angles, models are established with small-particle-sized (6.5 mm), medium-particle-sized (13 mm), and large-particle-sized (26 mm) debris flows with a total mass of 72 kg. The established DEM model of a small particle-sized debris flow impacting a flexible net is shown in Fig 2.

**Fig 3:**
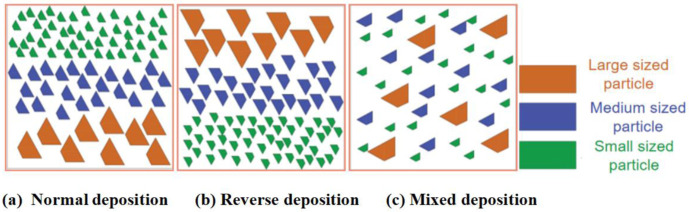
Initial accumulation states of debris flows with different particle sizes.

Different slopes result in different accumulation states. The total mass remains at 72 kg, and the mass ratio of small – particle – sized: medium – particle – sized: large – particle – sized is 40%: 30%: 30%. The accumulation states are normal deposition, reverse deposition, and mixed deposition, as shown in Fig 3.

## 3. Results and discussion

### 3.1. Velocity distribution of the debris flow impacting the flexible net

**Fig 4:**
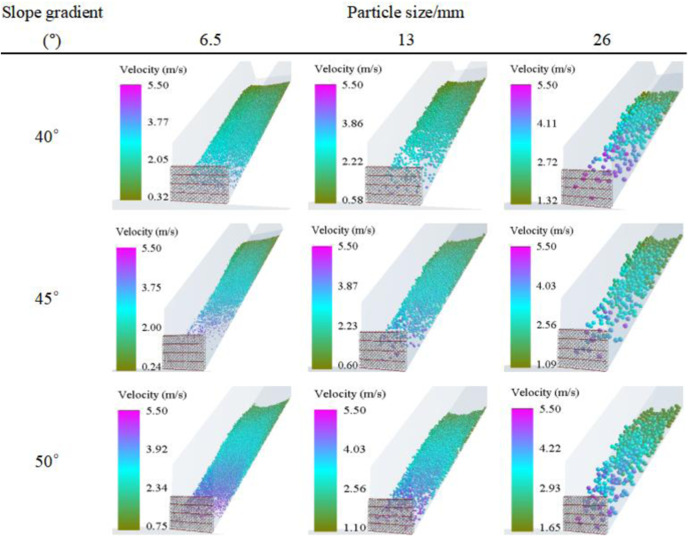
Velocity distribution of the debris flow when it initiates impact on the flexible net.

**Fig 5:**
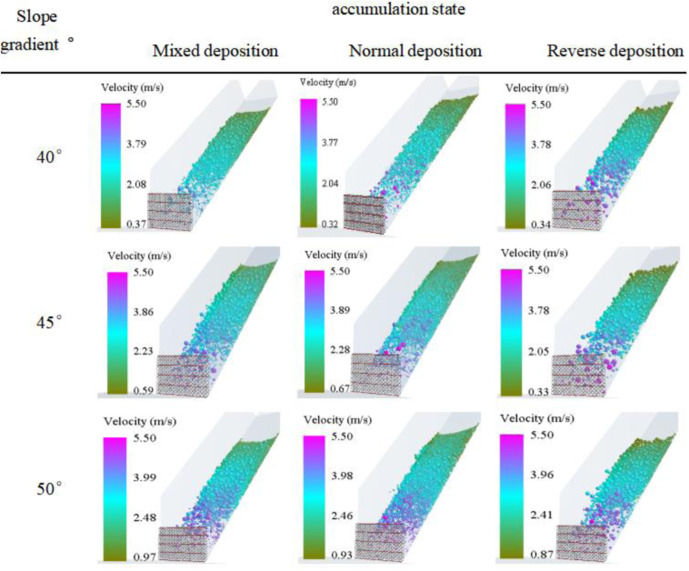
Velocity Distribution of Debris Flows with Different Accumulation States When Initiating Impacts on the Flexible Net.

Figs 4 and 5 present a detailed scenario of the velocity distribution of the debris flow impacting the flexible net. The maximum leading-edge velocity of small-sized particles (6.5 mm) is 5.39 m/s, whereas that of large-sized particles (26 mm) reaches 5.61 m/s. The slight increase in impact velocity with increasing particle size implies complex mechanical principles. Larger-sized particles possess greater masses, and during the acceleration process under the influence of gravity, they accumulate more kinetic energy. Consequently, they have a higher velocity when impacting the flexible net. From a microscopic perspective, there are relatively few collisions among large-sized particles, and they are less affected by interference factors such as air resistance. This allows them to maintain an accelerated state more smoothly, thus attaining a higher impact velocity.

The velocity distributions of debris flow in different accumulation states also merit in-depth exploration. In the mixed-accumulation state, owing to the intermingling of particles of different sizes, the collisions and frictions among them are intricate, resulting in a relatively uniform velocity distribution. In this state, the high-speed movement of large-sized particles is impeded by small-sized particles, whereas the low-speed movement of small-sized particles is driven by large-sized particles, resulting in a relatively balanced velocity state. In the normal-deposition and reverse-accumulation states, the velocity distribution significantly fluctuates. During normal deposition, the small-sized particles that impact first may be accelerated and pushed by subsequent large-sized particles, leading to substantial velocity changes. In reverse deposition, large-sized particles impact first, and their immense impact forces render the movement states of the surrounding small-sized particle complex, thereby increasing velocity fluctuations.

A higher impact velocity indicates greater momentum. According to the momentum theorem, the impact force on the flexible net will be greater. For example, in some debris-flow prevention and control projects in the southwestern mountainous regions of the United States, if a debris flow with large-sized particles and a high velocity strikes a flexible net, the instantaneous load borne by the flexible net may exceed its designed load-bearing capacity, causing the net to rupture or the support structure to be damaged. Therefore, when designing flexible nets, it is essential to fully consider the velocity distributions of debris flows with different particle sizes and accumulation states. Measures such as increasing the strength of the net ropes and optimizing the layout of the support structure should be adopted to ensure the safety of the protection structure. Moreover, in the selection of project sites and the design of early warning systems, these velocity distribution data should also be referred to, and evacuation routes for personnel and early warning times should be rationally planned to minimize disaster losses.

### 3.2. Tensile force of the support rope when the debris flow impacts the flexible net

**Fig 6:**
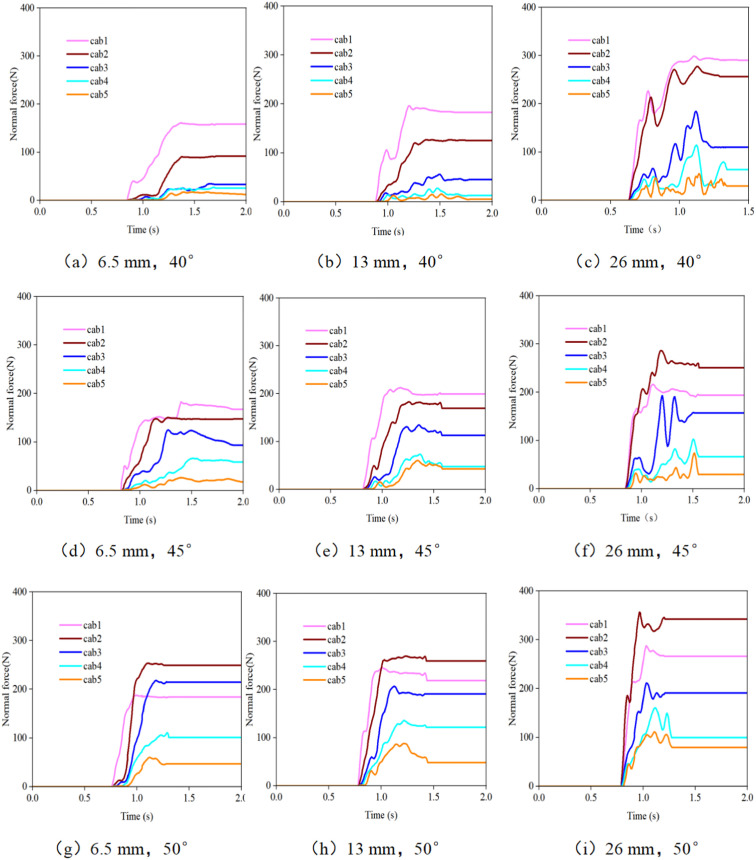
Time-history curve of the tensile force of the support rope when debris flows with different particle sizes impact the flexible net.

**Fig 7:**
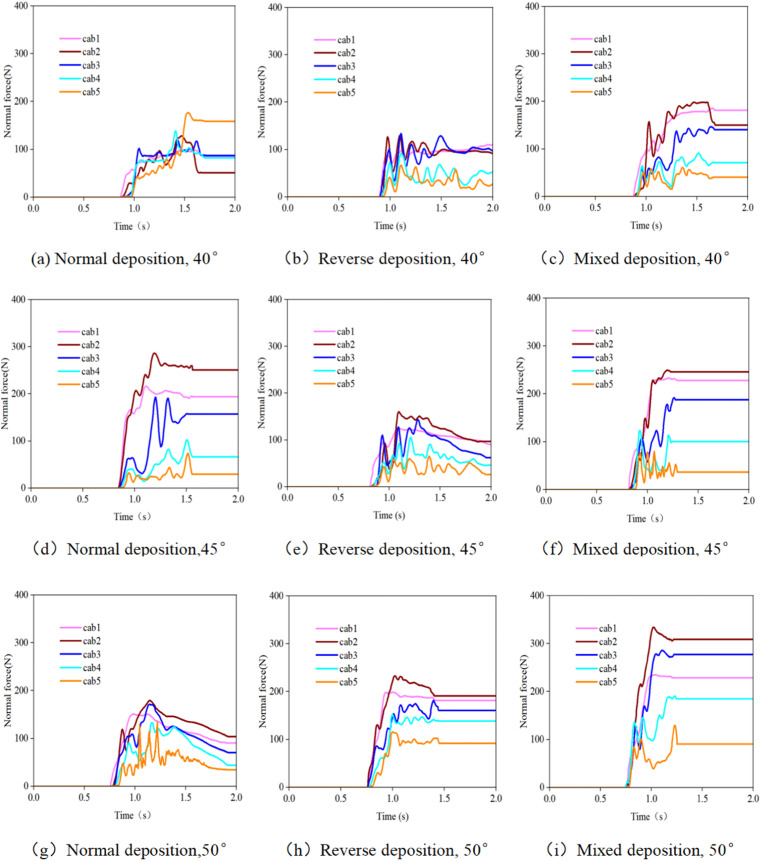
Time-history curve of the tensile force of the support rope when debris flows with different initial accumulation states impact the flexible net.

Figs 6 and 7 clearly illustrate the variation laws of the tensile force of the support rope under different particle sizes and accumulation states. The tensile force of the lower support ropes (Cab1 - Cab3) is significantly greater than that of the upper support ropes (Cab4 - Cab5). This phenomenon reveals the crucial position of the lower support ropes as the main load-bearing structures. During the impact process of a debris flow, due to the action of gravity, most of the debris flow accumulates downwards, subjecting the lower support ropes to more impact forces. From a mechanical perspective, the lower support ropes bear not only the direct impact force of the debris flow but also the tensile force transmitted due to the deformation of the net. Therefore, their stress conditions are more complex and intense.

As the particle size increases, the tensile force of the support rope significantly increases. Especially in the case of large particles (26 mm), the tensile force of the support rope reaches its maximum value. Large-sized particles carry more kinetic energy, and when they impact the flexible net, they generate a stronger instantaneous impact force on the support rope. Moreover, when large-sized particles are intercepted behind the net, they cause local stress concentration on the net, further increasing the tensile force of the support rope. For example, when a debris flow with 26 mm-sized particles impacts a flexible net, the tensile force borne by the support rope Cab1 may be several times greater than that when a debris flow with 6.5 mm-sized particles impacts. This poses extremely high requirements for the material strength and durability of the support rope.

In practical engineering, the tensile-force distribution of the support rope directly affects the stability and durability of the flexible net. If the strength of the lower support ropes is insufficient, they may break under the action of large tensile forces, thereby leading to failure of the entire flexible net structure. Thus, during the design process, attention should be focused on the stress conditions of the lower support ropes. High-strength and high-toughness materials, such as special alloy materials or steel wires with enhanced treatment, should be selected for manufacturing support ropes. Moreover, reasonably adjusting the mesh size according to the particle size of the debris flow is highly important. Smaller mesh sizes can more effectively intercept large debris flows, reducing their direct impact on the support ropes and thus decreasing the tensile-force fluctuations of the support ropes. In addition, measures such as increasing the number of support ropes and optimizing the connection method can be employed to increase the overall load-bearing capacity of the flexible net, ensuring its stable operation under various working conditions.

### 3.3. Accumulation height of debris flow after impacting the flexible net

Table 4 presents the differences in the accumulation heights of debris flows under different particle sizes and accumulation states. As the particle size increased, the accumulation height of the debris flow slightly increased. This is mainly because large-sized particles have a relatively large volume. Under the condition of the same mass, they occupy more space, resulting in an increase in the accumulation height. For example, when a debris flow with 26 mm-sized particles accumulates, since the volume of a single particle is several times greater than that of a 6.5 mm-sized particle, even with the same mass, its accumulation height is relatively greater. Moreover, the gaps between large-sized particles are relatively large, and it is difficult for them to be closely arranged during the accumulation process, which further increases the accumulation height.

**Table 4:** Accumulation heights of debris flows.

Debris flow (mm)	Deposition height (m)		
	40°	45°	50°
6.5	0.18	0.4	0.30
13	0.18	0.23	0.30
26	0.19	0.23	0.27
Normal deposition	0.40	0.40	0.39
Reverse deposition	0.37	0.38	0.38
Mixed deposition	0.26	0.31	0.38

The accumulation height is the greatest in the normal-accumulation state, whereas it is relatively small in the mixed-accumulation state. During normal deposition, small-sized particles can easily fill the gaps between large-sized particles, making the accumulation structure more compact and increasing the overall height. Conversely, in the mixed-accumulation state, particles of different sizes are intermingled, making it difficult to form a stable accumulation structure. Some particles may slide due to mutual extrusion, leading to a reduction in the accumulation height.

The accumulation height of the debris flow directly affects the deformation and damage of the flexible net. A higher accumulation height subjects the flexible net to greater pressure, causing excessive local deformation of the net. When the accumulation height exceeds a certain limit, the flexible net may experience stretching rupture, net rope breakage, and other problems. In some mountain-area protection projects, cases in which the bottom of the flexible net is crushed due to the excessive accumulation height of the debris flow have occurred, allowing a large amount of debris flow to rush into the protected area. Therefore, in practical engineering, the structure of the flexible net should be reasonably designed according to the accumulation height of the debris flow. Measures such as increasing the height of the net body and strengthening the bottom support structure can be taken to improve the bearing capacity of the flexible net against debris flows with high accumulation heights. Moreover, considering the onsite terrain and geological conditions, reasonable diversion or buffer areas should be set up to reduce the accumulation height of the debris flow in front of the flexible net, thereby reducing the pressure on the flexible net and ensuring the protection effect and stability of the net body.

### 3.4. Horizontal displacement of the support rope when the debris flow impacts the flexible net

**Fig 8:**
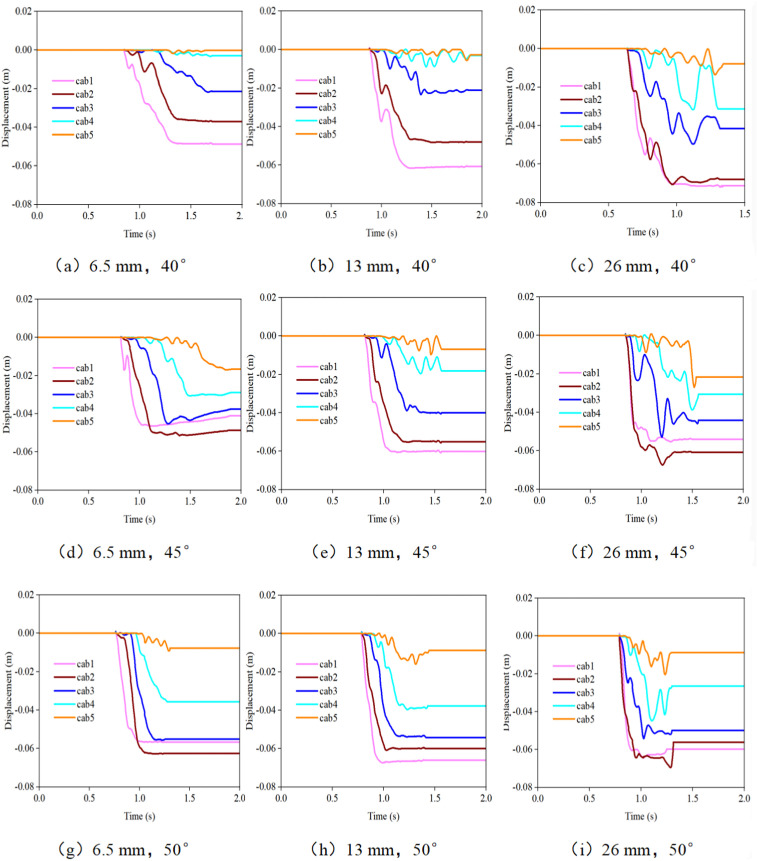
Time-history curves of the horizontal displacement of the support rope when debris flows with different particle sizes impact the flexible net.

**Fig 9:**
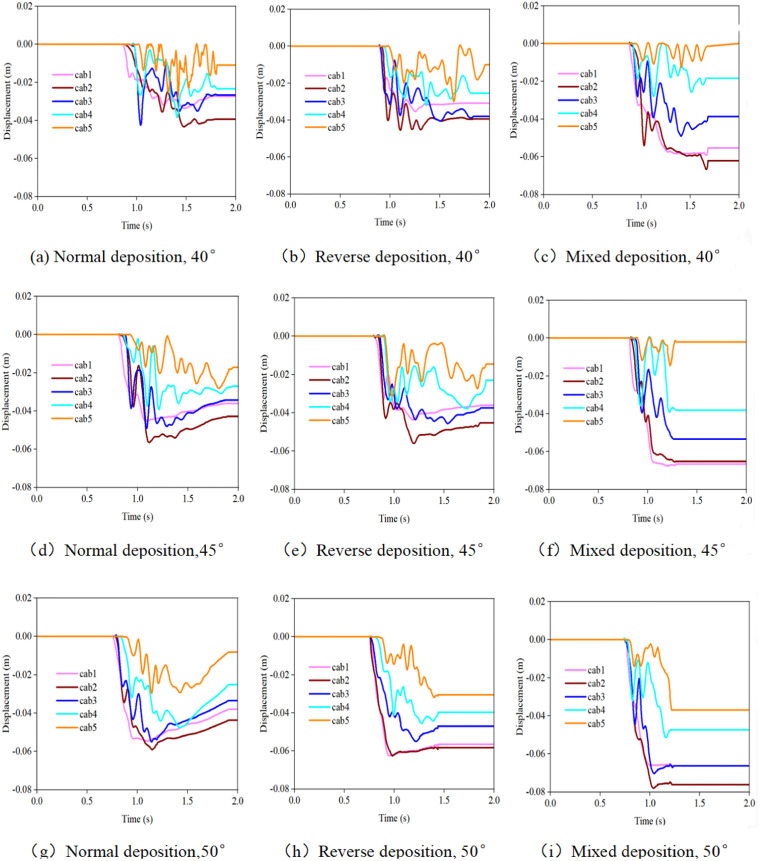
Time-history curves of the horizontal displacement of the support rope when debris flows with different initial accumulation states impact the flexible net.

Figs 8 and 9 display the changes in the horizontal displacement of the support rope under different working conditions. The horizontal displacement of the lower support ropes (Cab1 - Cab3) is relatively large, whereas that of the upper support ropes (Cab4 - Cab5) is relatively small. This is because the lower support ropes bear the main impact force of the debris flow and are more prone to deformation and displacement under the action of the force. When the debris flow impacts the flexible net, the impact force on the lower support ropes has not only a vertical component but also a horizontal component. These horizontal components push the support ropes to undergo horizontal displacement. In addition, while bearing a large tensile force, the deformation of the net body also drives the lower support ropes to move horizontally.

As the particle size increases, the horizontal displacement of the support rope significantly increases. When large-sized particles impact the flexible net, the high kinetic energy they carry causes greater deformation of the net body, resulting in an increase in the horizontal displacement of the support rope. For example, when a debris flow with 26 mm-sized particles impacts, the horizontal displacement of the support rope Cab1 may be several times larger than that when a debris flow with 6.5 mm-sized particles impacts. This is because large-sized particles have a stronger impact force, causing greater damage to the net body, making the deformation of the net body in the horizontal direction more obvious, and thus driving the support rope to produce a larger displacement.

The horizontal displacement of the support rope reflects the deformation of the flexible net when it is impacted by the debris flow. A large horizontal displacement may lead to excessive local deformation of the flexible net, affecting its protection effect. When the horizontal displacement of the support rope is too large, the shape of the net body changes, and the size and spacing of the mesh holes also change accordingly. This may allow debris flows that could originally be intercepted to pass through the net, reducing the protection performance. In practical engineering, attention should be focused on the horizontal displacement of the lower support ropes. By strengthening the connection strength between the support ropes and the fixed structure and adding auxiliary support structures such as diagonal cables, the horizontal displacement of the support ropes can be limited to ensure that their deformation is within a controllable range. Moreover, when designing flexible nets, the possible horizontal displacement of debris flows with different particle sizes should be fully considered, and a certain deformation space should be reserved to ensure that the net body can still maintain an effective protection function when subjected to impacts, improving the reliability and stability of the protection structure.

### 3.5. Accumulation morphology of debris flow and failure morphology of the flexible net

**Fig 10:**
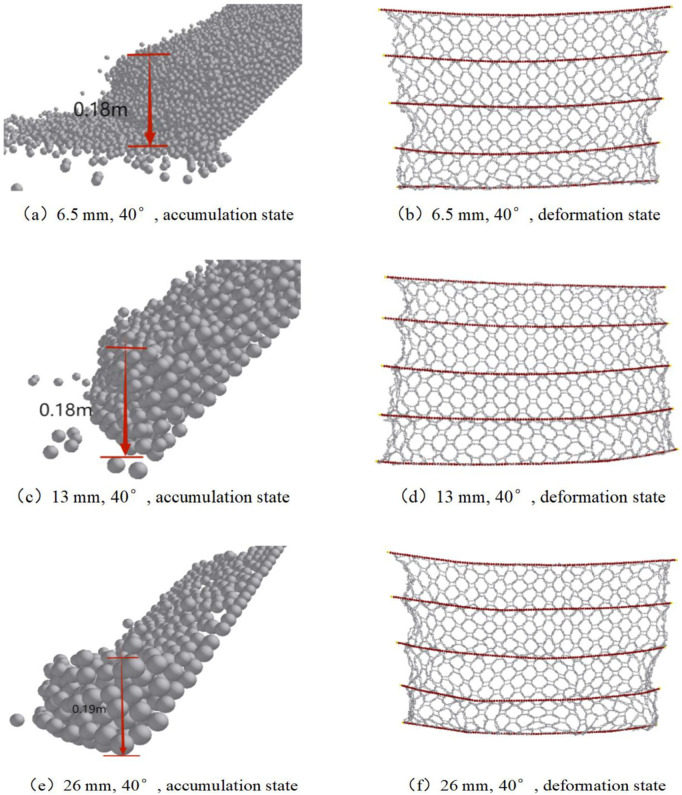
Front view of the accumulation state of the debris flow and the failure morphology of the flexible net after debris flow accumulation.

Fig 10 visually shows the differences in the accumulation morphology of debris flows with different particle sizes after impact with the flexible net and the failure morphology of the flexible net. Debris flows with small particles (6.5 mm) can partially pass through the net body because their particle size is smaller than the diameter of the mesh holes, and they have a certain degree of permeability during the impact process. This partial penetration phenomenon makes the impact force on the flexible net relatively dispersed, preventing excessive stress concentration in local areas and resulting in relatively small deformation of the flexible net. In actual protection scenarios, after small debris flows pass through a net, they may accumulate relatively uniformly behind the net, having a relatively small impact on the overall structure of the net.

However, debris flows with large-sized particles (26 mm) are completely intercepted behind the net. Since their particle size is larger than the diameter of the mesh holes, they cannot pass through the net. When these large-sized particles impact the net body, they transfer a large amount of kinetic energy to the net body, subjecting the flexible net to a very large impact force and thus causing greater deformation. When large-sized particles accumulate behind the net, they may form local accumulation peaks, further increasing the local stress on the net body and making the net body more prone to failure conditions such as stretching deformation and net rope breakage. For example, in some flexible net protection projects in mountainous areas, when large debris flows impact, obvious depressions and damage can often be observed on flexible nets at locations where large-sized particles accumulate.

The accumulation morphology of the debris flow and the failure morphology of the flexible net directly affect the service life and protection effect of the protection structure. In practical engineering, the mesh size of the flexible net should be reasonably designed according to the particle size distribution of the debris flow. In areas where the debris flow has small-sized and evenly distributed particles, the mesh size can be appropriately increased to improve the permeability of the flexible net, reduce the direct stress on the net body, and lower the failure risk. In areas where large debris flows are frequent, a flexible net with a smaller mesh size should be selected to ensure effective interception of the debris flow. Moreover, the strength and toughness of the net body should be increased to prevent damage to the net body due to excessive impact force. In addition, regular inspection and maintenance of the flexible net and timely repair or replacement of damaged parts according to the accumulation morphology and failure conditions are crucial for ensuring the long-term stable operation of the protection structure. By comprehensively considering these factors, the design and use of flexible nets can be optimized, their protection ability against debris-flow disasters can be improved, and disaster losses can be minimized.

### 3.6. Discussion on the potential use of DEM simulations

DEM simulations offer transformative potential in debris flow protection by resolving particle-scale dynamics to optimize structural resilience. The study reveals critical insights: non-uniform tensile forces (60–70% concentrated in lower ropes) necessitate localized reinforcement, while particle size-dependent velocity amplification informs permeability selection to balance interception efficiency and durability. DEM-derived thresholds for critical particle size and accumulation height enable adaptive net designs tailored to regional flow characteristics, minimizing over-engineering. Integration with machine learning enhances predictive accuracy for multi-phase flows, and GPU-accelerated solvers address computational constraints. By linking granular interactions to macroscale performance, DEM facilitates risk-informed designs-predicting failure modes (e.g., anchor slippage) and guiding real-time mitigation strategies. This paradigm shift advances cost-effective, resilient protection systems, bridging theoretical granular mechanics with actionable engineering solutions for mountainous regions.

## 4. Conclusions

This paper addresses the frequent occurrence of debris flow disasters in mountainous areas and the insufficient actual protection effect of flexible protection nets. Through discrete element numerical (DEM) simulation, the dynamic behavior of debris flows with different flow states impacting flexible nets is systematically studied, with a focus on analysing the influence mechanism of flexible-net permeability on debris-flow impact loads. The following conclusions are obtained:

[1] The particle size of a debris flow affects the impact velocity, and larger-sized particles have a higher velocity. For example, the maximum front edge velocity of a debris flow with 6.5 mm-sized particles is 5.39 m/s, whereas that of a debris flow with 26 mm-sized particles is 5.61 m/s. The velocities vary with different accumulation states. This law should be considered when designing flexible nets to ensure safety.
[2] The lower support ropes of the flexible net bear a large load, and the tensile force increases as the particle size of the debris flow increases. Taking a slope of 40° as an example, when impacted by 6.5 mm-sized particles, the tensile force of Cab1 is 161.3 N, and when impacted by 26 mm-sized particles, it reaches 302.0 N. In the design, measures such as strengthening the lower support should be taken to improve stability.
[3] The accumulation height of a debris flow is affected by the particle size and accumulation state. An excessively high accumulation height can damage the flexible net. For example, on a 45° slope, the accumulation height of a debris flow with 6.5 mm-sized particles is 0.4 m, whereas that of a debris flow with 26 mm-sized particles is 0.23 m. The normal depositional state has a greater accumulation height than the mixed depositional state. In engineering, the net-body structure should be optimized.
[4] Small debris flows cause less deformation of the flexible net, whereas large debris flows cause more deformation. The 6.5 mm-sized particles can partially pass through the net, whereas the 26 mm-sized particles are completely intercepted. The mesh size should be set according to the particle size, and regular maintenance should be carried out to improve the protection ability.
[5] This research was supported by the project of scientific and technological talents of ordinary higher education institutions in Guizhou Provence (QJHKY[2022]055), the Natural Science Foundation of Guizhou Province(Grant No. Qiankehe Foundation-ZK[2022] General 533), the National NaturalScience Foundation of China (Grant No. 42367026) and the Guizhou expressway group Co., Ltd. science and technology project (2023-GS004).
